# Supplementary material for: Predictive Modeling of Long-Term Care Needs in Traumatic Brain Injury Patients Using Machine Learning
Source: Diagnostics (Basel). 2024 Dec 25;15(1):20. doi: 10.3390/diagnostics15010020 (PMC11720696; doi:10.3390/diagnostics15010020)
Supplement: Supplementary file 1 [file diagnostics-15-00020-s001.zip › Supplemental Table S1 RTable. Statistics of missing value for model training.pdf]

Supplemental Table S1

SRTable. Statistics of missing value for model training.

| Variable | Number of missing values | % of total |
|---|---|---|
| SOFA | 862 | 24.55% |
| FiO2 | 384 | 10.94% |
| Pupil size | 265 | 7.55% |
| Hight | 175 | 4.98% |
| APACHE II | 67 | 1.91% |
| Weight | 28 | 0.80% |
| DBP | 8 | 0.23% |
| Muscle power | 7 | 0.20% |
| Mean Arterial Pressure(MAP) | 6 | 0.17% |
| Glasgow Coma Scale(GCS) | 2 | 0.06% |
| Pupil reflex | 2 | 0.06% |
| Body temperature(BT) | 2 | 0.06% |
