# Supplementary material for: Predictive Modeling of Long-Term Care Needs in Traumatic Brain Injury Patients Using Machine Learning
Source: Diagnostics (Basel). 2024 Dec 25;15(1):20. doi: 10.3390/diagnostics15010020 (PMC11720696; doi:10.3390/diagnostics15010020)
Supplement: Supplementary file 1 [file diagnostics-15-00020-s001.zip › Supplemental Table S2. Hyper-parameters range for experiments.pdf]

Supplemental Table S2. Hyper-parameters range for experiments

Method and Hyper-parameter	Feature=44	Feature=27	Feature=18	Feature=11
Logistic regression				
penalty	l1, l2	l1, l2	l1, l2	l1, l2
C	1e-5, 1e-4, 1e-3, 1e-2, 1, 5, 10, 30	1e-4, 1e-3, 1e-2, 1, 5, 10, 15	1e-4, 1e-3, 1e-2, 1, 5, 15	1e-4, 1e-3, 1e-2, 1, 5
max_iter	15, 30, 50, 75, 100	30, 50, 75, 100	30, 50, 75, 100	30, 50, 75, 100
Defaults were used for other parameters	dual=False, tol=0.0001, fit_intercept=True, intercept_scaling=1, class_weight=None, random_state=None, solver='lbfgs', multi_class='auto', verbose=0, warm_start=False, n_jobs=None, l1_ratio=Non			
Random forest				
random_state	15, 33, 42	15, 25, 33, 50	15, 33, 42	15, 33, 42
n_estimators	100, 250, 500, 750	50, 100, 150, 300, 500	30, 50, 100, 150, 500	50, 100, 150, 200, 250
max_depth	7,15, 30, 45, 50, 100	3, 5, 15, 25	7,15, 25, 30	7,15, 25
min_samples_split	2, 5 10, 15	5, 10, 15	5, 10, 15, 20	10, 15, 20
max_features	auto, sqrt, 0.5, 1.0, 1.5	auto, sqrt, 0.5, 1.0, 2.0	auto, sqrt, 0.5, 1.0	auto, sqrt, 0.5, 1.0
Defaults were used for other parameters	criterion='gini', min_samples_leaf=1, min_weight_fraction_leaf=0.0, max_leaf_nodes=None, min_impurity_decrease=0.0, bootstrap=True, oob_score=False, n_jobs=None, verbose=0, warm_start=False, class_weight=None, ccp_alpha=0.0, max_samples=None			
LightGBM				
learning_rate	1e-1, 1e-2	1e-1, 1e-2, 0.005	1e-1, 1e-2, 1e-3	1e-1, 1e-2, 1e-3
n_estimators	100, 250, 500, 750	50, 90, 100, 150	100, 150, 200, 500	100, 150, 200, 500
max_depth	10, 15, 30, 50, 100	15, 30, 50	15, 30, 50	15, 30, 50
Defaults were used for other parameters	boosting_type='gbdt', num_leaves=31, subsample_for_bin=200000, objective=None, class_weight=None, min_split_gain=0.0, min_child_weight=0.001, random_state=None, min_child_samples=20, subsample=1.0, subsample_freq=0, colsample_bytree=1.0, reg_alpha=0.0, reg_lambda=0.0, n_jobs=None, importance_type='split'			
XGBoost				
learning_rate	1e-3, 1e-2, 1e-1	1e-3, 1e-2, 1e-1	1e-3, 1e-2, 1e-1	1e-3, 1e-2, 1e-1
gamma	1e-2, 1e-3, 1e-4	0.2, 1e-1, 1e-2, 1e-3	0.2, 0.1, 0.01	0.2, 0.1, 0.01
n_estimators	200, 500, 750, 900, 1000	100, 150, 200, 500	100, 150, 200, 250, 500	150, 250, 500
max_depth	3, 15, 25, 30, 50	15, 25, 30	15, 25, 30, 35	15, 25, 30
Defaults were used for other parameters	Objective='binary:logistic', n_estimators: int = 100, verbosity=0, booster='gbtree', tree_method='auto', n_jobs=1, gamma=0, min_child_weight=1, ax_delta_step=0,subsample=1, colsample_bytree=1, colsample_bylevel =1, colsample_bynode=1, reg_alpha=0, reg_lambda=0, scale_pos_weight=1, base_score=0.5, random_state=0, missing=None, monotone_constraints=None,			

Method and Hyper-parameter	Feature=44	Feature=27	Feature=18	Feature=11
	interaction_constraints=None, importance_type=None, gpu_id=None, validate_parameters=False, predictor='auto', enable_categorical=False			
MLP				
hidden_layer_sizes	(250,65), (200,), (100,), (90,60)	(200,), (150,75), (100,), (90,)	(200,), (150,75,), (100,), (90,)	(200,), (100,), (100,42)
max_iter	200, 100, 50, 15, 10	100, 50, 15, 10	50, 25, 15, 10	50, 25, 15, 10
learning_rate_init	1e-3, 1e-2, 1e-1	1e-2, 1e-1	1e-2, 1e-1	1e-2, 1e-1
early_stopping	True, False	True, False	True, False	True, False
random_state	55, 33, 15, 5	54, 50, 33, 20	55, 50, 33, 20, 5	33, 20, 15, 5
alpha	1e-4, 1e-5	1e-4, 1e-5	1e-4, 1e-5	1e-4, 1e-5
Defaults were used for other parameters	activation='relu', solver='adam', batch_size='auto', learning_rate='constant', power_t=0.5, shuffle=True, tol=0.0001, verbose=False, warm_start=False, momentum=0.9, nesterovs_momentum=True, validation_fraction=0.1, beta_1=0.9, beta_2=0.999, epsilon=1e-08, n_iter_no_change=10, max_fun=15000			

The hyper-parameters that are not described in this table are set to the default values used in the scikit-learn library

Grid search with 5-fold cross-validation for hyper-parameters tuning for each algorithm was conducted for obtaining optimal model
