# Supplementary material for: Predictive Modeling of Long-Term Care Needs in Traumatic Brain Injury Patients Using Machine Learning
Source: Diagnostics (Basel). 2024 Dec 25;15(1):20. doi: 10.3390/diagnostics15010020 (PMC11720696; doi:10.3390/diagnostics15010020)
Supplement: Supplementary file 1 [file diagnostics-15-00020-s001.zip › Supplemental Table S3. Training dataset 5-fold cross-validation results.pdf]

Supplemental Table S3 Training dataset 5-fold cross-validation results (mean ± standard deviation).

(A)

Algorithm (44)	Accuracy	Sensitivity	Specificity	AUC
Logistic Regression	0.732 ± 0.024	0.665 ± 0.038	0.800 ± 0.011	0.807 ± 0.022
Random Forest	0.901 ± 0.050	0.922 ± 0.119	0.881 ± 0.021	0.975 ± 0.031
LightGBM	0.920 ± 0.108	0.884 ± 0.230	0.955 ± 0.016	0.983 ± 0.033
XGBoost	0.861 ± 0.035	0.958 ± 0.083	0.763 ± 0.031	0.972 ± 0.043
MLP	0.799 ± 0.020	0.878 ± 0.055	0.721 ± 0.035	0.857 ± 0.017
Stacking	0.906 ± 0.085	0.884 ± 0.200	0.927 ± 0.035	0.978 ± 0.033

(B)

Algorithm (27)	Accuracy	Sensitivity	Specificity	AUC
Logistic Regression	0.705 ± 0.015	0.621 ± 0.018	0.788 ± 0.016	0.798 ± 0.018
Random Forest	0.829 ± 0.036	0.896 ± 0.071	0.762 ± 0.018	0.913 ± 0.041
LightGBM	0.884 ± 0.075	0.869 ± 0.171	0.899 ± 0.026	0.956 ± 0.055
XGBoost	0.790 ± 0.018	0.967 ± 0.066	0.613 ± 0.057	0.968 ± 0.043
MLP	0.765 ± 0.018	0.858 ± 0.058	0.672 ± 0.075	0.840 ± 0.013
Stacking	0.856 ± 0.065	0.873 ± 0.179	0.839 ± 0.080	0.956 ± 0.042

(C)

Algorithm (18)	Accuracy	Sensitivity	Specificity	AUC
Logistic Regression	0.692 ± 0.010	0.592 ± 0.005	0.792 ± 0.017	0.783 ± 0.020
Random Forest	0.887 ± 0.059	0.912 ± 0.126	0.861 ± 0.016	0.969 ± 0.038
LightGBM	0.870 ± 0.046	0.894 ± 0.098	0.845 ± 0.014	0.939 ± 0.041
XGBoost	0.868 ± 0.079	0.888 ± 0.222	0.849 ± 0.067	0.977 ± 0.044
MLP	0.772 ± 0.025	0.787 ± 0.080	0.757 ± 0.046	0.845 ± 0.020
Stacking	0.874 ± 0.076	0.871 ± 0.190	0.877 ± 0.044	0.962 ± 0.037

(D)

Algorithm (11)	Accuracy	Sensitivity	Specificity	AUC
Logistic Regression	0.703 ± 0.015	0.613 ± 0.016	0.792 ± 0.016	0.785 ± 0.021
Random Forest	0.876 ± 0.050	0.918 ± 0.113	0.833 ± 0.020	0.956 ± 0.038
LightGBM	0.850 ± 0.039	0.889 ± 0.089	0.811 ± 0.023	0.923 ± 0.039
XGBoost	0.859 ± 0.074	0.903 ± 0.190	0.814 ± 0.047	0.968 ± 0.057
MLP	0.768 ± 0.027	0.840 ± 0.046	0.696 ± 0.074	0.829 ± 0.025
Stacking	0.855 ± 0.074	0.884 ± 0.184	0.826 ± 0.042	0.952 ± 0.039
