# Supplementary figures and images for: Predictive Modeling of Long-Term Care Needs in Traumatic Brain Injury Patients Using Machine Learning
Source: Diagnostics (Basel). 2024 Dec 25;15(1):20. doi: 10.3390/diagnostics15010020 (PMC11720696; doi:10.3390/diagnostics15010020)

Supplemental Figure S1. Learning curve for 11-feature Random Forest model

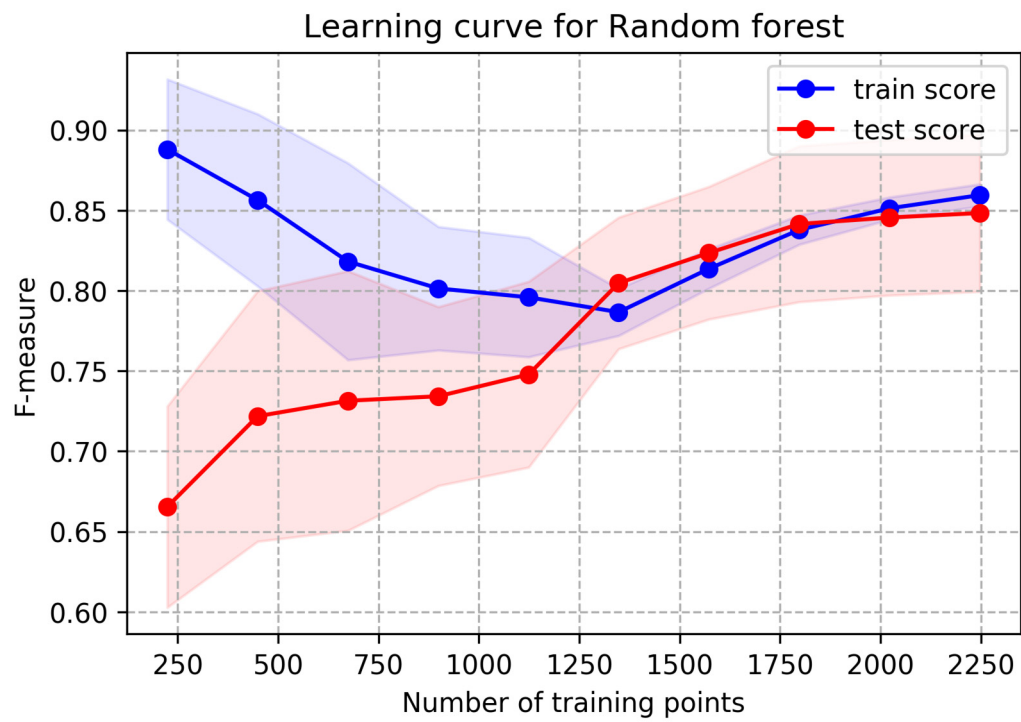

Supplement: Supplementary file 1 [file diagnostics-15-00020-s001.zip › Supplemental Figure S1. Learing curve for 11-feature Random Forest model (1).pdf]
